# Prothrombotic factors do not increase the risk of recurrent ischemic events after cryptogenic stroke at young age: the FUTURE study

**DOI:** 10.1007/s11239-018-1631-4

**Published:** 2018-02-26

**Authors:** Mijntje M. I. Schellekens, Mayte E. van Alebeek, Renate M. Arntz, Nathalie E. Synhaeve, Noortje A. M. M. Maaijwee, Hennie C. Schoonderwaldt, Maureen J. van der Vlugt, Ewoud J. van Dijk, Loes C. A. Rutten-Jacobs, Frank-Erik de Leeuw

**Affiliations:** 1 Department of Neurology, Donders Institute for Brain, Cognition and Behaviour, Center for Neuroscience, RadboudUMC, PO Box 9101, 6500 HB Nijmegen, The Netherlands; 2 Department of Neurology, Elisabeth Tweesteden Hospital, PO Box 90151, 5000 LC Tilburg, The Netherlands; 3 Center for Neurology and Neurorehabilitation, Luzern State Hospital, Spitalstrasse 31, 6000 Luzern 16, Switzerland; 4 Department of Cardiology, RadboudUMC, PO Box 9101, 6500 HB Nijmegen, The Netherlands; 5 Department of Clinical Neurosciences, University of Cambridge, Cambridge, UK

**Keywords:** Blood coagulation factors, Infection, Prognosis, Risk factors, Stroke

## Abstract

**Background:**

The role of hypercoagulable states and preceding infections in the etiology of young stroke and their role in developing recurrent ischemic events remains unclear. Our aim is to determine the prevalence of these conditions in patients with cryptogenic stroke at young age and to assess the long-term risk of recurrent ischemic events in patients with and without a hypercoagulable state or a recent pre-stroke infection with Borrelia or Syphilis.

**Patients and methods:**

We prospectively included patients with a first-ever transient ischemic attack or ischemic stroke, aged 18–50, admitted to our hospital between 1995 and 2010. A retrospective analysis was conducted of prothrombotic factors and preceding infections. Outcome was recurrent ischemic events.

**Results:**

Prevalence of prothrombotic factors did not significantly differ between patients with a cryptogenic stroke and with an identified cause (24/120 (20.0%) and 32/174 (18.4%) respectively). In patients with a cryptogenic stroke the long-term risk [mean follow-up of 8.9 years (SD 4.6)] of any recurrent ischemic event or recurrent cerebral ischemia did not significantly differ between patients with and without a hypercoagulable state or a recent infection. In patients with a cryptogenic stroke 15-years cumulative risk of any recurrent ischemic event was 24 and 23% in patients with and without any prothrombotic factor respectively.

**Conclusions:**

The prevalence of prothrombotic factors and preceding infections did not significantly differ between stroke patients with a cryptogenic versus an identified cause of stroke and neither is significantly associated with an increased risk of recurrent ischemic events after cryptogenic stroke.

**Electronic supplementary material:**

The online version of this article (10.1007/s11239-018-1631-4) contains supplementary material, which is available to authorized users.

## Background

Up to 12% of all strokes occur in adults aged between 18 and 50 years [1]. Each year about 2 million young people suffer a stroke worldwide, with absolute numbers that continue to increase [2]. In about two-thirds of all young adults the cause of stroke is identified after routine evaluation and can be classified according to the TOAST classification as due to large artery disease, small vessel disease, a cardio-embolic source or another determined cause [3]. In the remaining one-third of all patients no cause is identified after routine examination and they are classified as those with a cryptogenic cause. In those patients, the treating physician can order numerous tests to disclose the cause of stroke ranging from coagulation panels, autoimmune parameters or markers of infection. However, the level of evidence for a causal role of these factors in the etiology of stroke usually does not exceed the level of an expert opinion. First, differences in the prevalence of aberrant values between patients with a stroke at young age and the healthy population have usually not reliably been assessed [4–8]. Second, proof of causality, that would be supported by a temporal relation between an aberrant factor (risk factor) and a long-term increased risk of incident (cerebro)vascular disease, has never clearly been demonstrated as long-term prospective studies are scarce in this field [9–14].

The aim of the present study is therefore to investigate the prevalence of prothrombotic factors and that of a recent preceding infection with Borrelia burgdorferi or Treponema pallidum (included in our current Young Stroke protocol) in patients with a cryptogenic stroke. Furthermore, in order to assess causality between these presumptive risk factors and stroke, the long-term risk of recurrent ischemic events in these patients with and without a hypercoagulable state or a recent infection was assessed.

## Patients and methods

### Patients and study design

This study is a part of the FUTURE (Follow-Up of Transient ischemic attack and stroke patients and Unelucidated Risk factor Evaluation) study, a prospective cohort study on risk factors and prognosis of ischemic and hemorrhagic stroke in young adults [15]. Details of the study have been described elsewhere [16]. The Medical Review Ethics Committee region Arnhem-Nijmegen approved the study. All participants signed an informed consent.

To minimize bias resulting from changing diagnostic techniques, the World Health Organization definitions for transient ischemic attack (TIA) and stroke were used [17, 18]. TIA was defined as a rapidly evolving focal neurological deficit, with vascular cause only, lasting less than 24 h. Stroke was defined as focal neurologic deficit persisting for more than 24 h [17, 18].

Patients were identified through a prospective registry of all consecutive patients with a young stroke that has been maintained at our centre with a standardized data collection of baseline characteristics. A retrospective registry analysis was conducted of prothrombotic factors and preceding infections from this prospective cohort.

In the present study, we included all patients with a first-ever TIA or ischemic stroke, aged 18–50, admitted to the Radboud University Medical Centre Nijmegen from 1995 till 2010. Due to the changes in standard diagnostic procedures over time, the completeness of the laboratory data varied among patients. Since 1995, patients most often were diagnosed and treated according to a standardized and structured ‘young stroke protocol’ with, as a result, more complete data from that time on.

Cardiovascular risk factors at baseline were defined as either a history of a risk factor (mentioned in medical history or the use of medication) or detected during admission or analysis of the stroke [19].

Assessment of the etiology (modified Trial of ORG 10,172 in Acute Stroke Treatment (TOAST) classification) [20] and the stroke severity [National Institutes of Health Stroke Scale (NIHSS)] [21] was performed retrospectively for all cases by a validated approach [22, 23]. Compared to the original TOAST classification,[24] the presently used classification has an additional category ‘likely large-artery atherosclerosis’ [20].

According to Saver, routine evaluation included patients’ history taking, physical examination, brain imaging, vessel-imaging, evaluation of cardiac rhythm and structure and standard hematologic testing [25]. Patients with a cryptogenic stroke after routine evaluation will be further referred to as cryptogenic.

### Laboratory investigations

Laboratory data on prothrombotic factors and infectious pathogens were retrieved from medical records when available. Testing for prothrombotic factors included: protein C activity, free protein S antigen, antithrombin III activity, factor V Leiden mutation, factor II mutation (prothrombin G20210A mutation), factor VIII activity, anticardiolipin antibodies (IgG and IgM) and lupus anticoagulant. Testing for lupus anticoagulant took a two-step approach. Screening was done with the dilute Russells viper venom time (dRVVT) or the activated partial thromboplastin time (aPTT). When positive (according local laboratory protocols) confirmation was sought with dRVVT and/or the Staclot-LA test. Lupus anticoagulant was considered positive if either one of these two confirmatory tests was abnormal. Infection screening included: Borrelia burgdorferi (IgG and IgM) and Treponema pallidum (i.e. syphilis). Screening for syphilis (for infection with Treponema pallidum) was done according to a two step approach. First serum was screened for the presence of Treponema pallidum antibodies using an enzyme-linked assay (ELISA).When positive, a confirmatory test by means of a Treponema pallidum Particle Agglutination (TPPA) test was performed. In case of a discrepancy (positive ELISA and negative TPPA) an immunoblot was used. For all measurements cut-off values were based on local laboratory protocols, except for factor VIII activity, for which an increased activity of greater than 200% was used as cut-off. Coagulation abnormalities were defined as follows: protein C activity lower than 70%, free protein S antigen lower than 65 and 55% for men and women respectively, antithrombin III activity lower than 85%. Blood samples collected within 180 days from the index event were included in the present study. Confirmatory testing was not performed for all positive laboratory tests.

Because of the small number of patients with a single prothrombotic factor, composite variables were made for patients with any prothrombotic factor or any positive antiphospholipid antibody (aPL) (patients who were positive for lupus anticoagulant, or anticardiolipin (IgG or IgM) or any combination of these).

### Follow-up

Follow-up assessment took place from 2009 to 2012 and subsequently from August 2014 to January 2015. In case information from the last follow-up assessment was missing, data from the previous follow-up were used [19]. Patients alive underwent a structured interview to evaluate for recurrent events. In case a patient had died, this information was retrieved from the general practitioner by structured questionnaires. When patients or their general practitioner confirmed that the patient had a recurrent event after their index event, medical records were retrieved from their treating physicians and verified by a neurologist or cardiologist. Recurrent cerebral ischemia (TIA or ischemic stroke) was defined similar to the index event, myocardial infarction was defined according to the universal definition of myocardial infarction [26].

### Outcome

Outcome was the occurrence of any recurrent ischemic event, defined as the composite event of cerebral ischemia and other arterial events (myocardial infarction, coronary artery bypass grafting, percutaneous coronary intervention, carotid endarterectomy or peripheral artery revascularisation procedures).

### Statistical analysis

Baseline characteristics were compared between participants and non-participants. The prevalence of prothrombotic factors and infectious pathogens was compared between patients with a cryptogenic stroke and patients with an identified cause of stroke. In addition prothrombotic factors and infectious pathogens were stratified by TOAST-subtype. Independent-samples t-test, Mann–Whitney U test or Fisher’s exact test were used when appropriate.

Next, the cumulative risk of any recurrent ischemic event and recurrent cerebral ischemia for patients with a cryptogenic stroke was estimated with Kaplan–Meier survival analysis, stratified by any prothrombotic factor and any aPL. Patients who died and/or did not reach the endpoint were censored. Person-years at risk were calculated for each patient from date of index event until recurrent event, death or end of follow-up. Survival plots were curtailed at 15 years to ensure that the provided survival plots were reliable for all subgroups [27].

Univariate cox proportional hazard analysis was used to calculate hazard ratios (HR) for the risk of any recurrent ischemic event or recurrent cerebral ischemia for the individual prothrombotic factors, infectious pathogens and the composed variables (e.g. any prothrombotic factor and any aPL). All p-values less than 0.05 were considered as significant. IBM SPSS Statistics 22 was used for all statistical analysis.

## Results

### Study population

We identified 562 patients who were eligible to the current study. From this group, 48 declined study participation and 99 were excluded because of loss to follow-up, leaving 415 patients with a first-ever TIA (n = 150) or ischemic stroke (n = 265) in this study. No differences in baseline characteristics between participants and the excluded patients (patients who refused (n = 48) or were lost to follow-up (n = 99)) were present with respect to type of index event, sex, age at index event or NIHSS at admission.

After routine evaluation 156 (37.6%) patients had a cryptogenic stroke, of whom 141 (90.4%) had an unknown cause and 15 (9.6%) had an ‘other defined’ cause after complete evaluation. Baseline characteristics are presented in Table 1. Mean follow-up duration of the total study population was 8.5 (SD 4.6) years and of the patients with a cryptogenic stroke 8.9 (SD 4.6) years.

**Table 1:** Baseline characteristics

	Total	Cryptogenic
n (% of total)	415 (100)	156 (37.6)
Mean age at event, years (SD)	40.9 (7.6)	39.6 (8.0)
Men, n (%)	180 (43.4)	70 (44.9)
Stroke subtype		
TIA	150 (36.1)	69 (44.2)
Ischemic stroke	265 (63.9)	87 (55.8)
Mean follow-up, years (SD)	8.5 (4.6)	8.9 (4.6)
TOAST		
Large artery disease	22 (5.3)	–
Likely large artery disease	54 (13.0)	–
Cardio-embolic stroke	59 (14.2)	–
Small vessel disease	49 (11.8)	–
Other defined	76 (18.3)	15 (9.6)
Multiple causes	14 (3.4)	–
Unknown	141 (34.0)	141 (90.4)
Median NIHSS at admission (IQR)	2 (0–5)	1 (0–5)
History of cardiovascular risk factors		
Diabetes	34 (8.2)	3 (1.9)
Hypertension	143 (34.5)	18 (11.5)
Dyslipidemia^a^	303 (82.1)	107 (76.4)
Atrial fibrillation	4 (1.0)	0 (0)
Smoking^a^	177 (44.3)	52 (34.2)
Excess drinking	22 (5.3)	6 (3.8)

*SD* standard deviation; *TIA* transient ischemic attack; *TOAST* Trial of ORG 10,172 in Acute Stroke Treatment; *NIHSS* National Institute of Health Stroke Scale; *IQR* interquartile range

^a^Missing data: dyslipidemia 11.1%; smoking 3.6%

### Laboratory data

The presence of prothrombotic factors and infectious pathogens stratified by patients with an identified cause and patient with a cryptogenic stroke is shown in Table 2. Screening for at least 1 prothrombotic factor or aPL in patients with a cryptogenic stroke took place in 120 (76.9%) and 117 (75.0%) respectively. In patients with an identified cause screening for at least 1 prothrombotic factor or aPL took place in 174 (67.2%) and 167 (64.5%) respectively. All prothrombotic factors and aPLs were determined in 48 (30.8%) and 95 (60.9%) of the patients with a cryptogenic stroke respectively. Any prothrombotic factor was found in 24 (20.0%) patients with a cryptogenic stroke and any positive aPL in 7 (6.0%). In patients with an identified cause of stroke any prothrombotic factor and any aPL was found in 32 (18.4%) en 10 (6.0%) patients respectively. There were no significant differences in proportions of prothrombotic factors or infectious pathogens between patients with a cryptogenic stroke and patients with an identified cause of stroke. A non-significant higher proportion of patients heterozygous for factor V was found in patients with a cryptogenic stroke (8.1% versus 2.5%). Online Supplementary Table I shows the distribution of prothrombotic factors and infectious pathogens stratified by TOAST category.

**Table 2:** Distribution of prothrombotic factors and recent infections

	Positive result/n_investigated_ (%)	
	Cause indentified	Cryptogenic
Prothrombotic factors		
Protein C activity	4/144 (2.8)	3/111 (2.7)
Free protein S antigen	3/142 (2.1)	3/110 (2.7)
Antithrombin III activity	11/154 (7.1)	4/108 (3.7)
Factor V mutation	3/121 (2.5)	7/86 (8.1)
Factor II mutation	2/111 (1.8)	1/78 (1.3)
Factor VIII activity	6/100 (6.0)	4/57 (7.0)
Lupus anticoagulant	5/152 (3.3)	2/110 (1.8)
Anticardiolipin IgG	3/146 (2.1)	3/103 (2.9)
Anticardiolipin IgM	3/146 (2.1)	4/102 (3.9)
Any prothrombotic factor	32/174 (18.4)	24/120 (20.0)
Any aPL	10/167 (6.0)	7/117 (6.0)
Infectious pathogens		
Borrelia IgG	2/141 (1.4)	1/98 (1.0)
Borrelia IgM	3/141 (2.1)	2/98 (2.0)
Syphilis	0/149 (0)	1/100 (1.0)

*aPL* antiphospholipid antibody

### Recurrent ischemic events

During 1393 person-years of follow-up in patients with a cryptogenic stroke, any recurrent ischemic event was recorded in 29 (18.6%) patients, of whom 25 (86.2%) suffered recurrent cerebral ischemia. In 22 (75.9%) of these patients with any recurrent ischemic event screening for at least 1 prothrombotic factor took place. Of them only 3 (13.6%) patients had at least 1 prothrombotic factor.

**Fig. 1:**
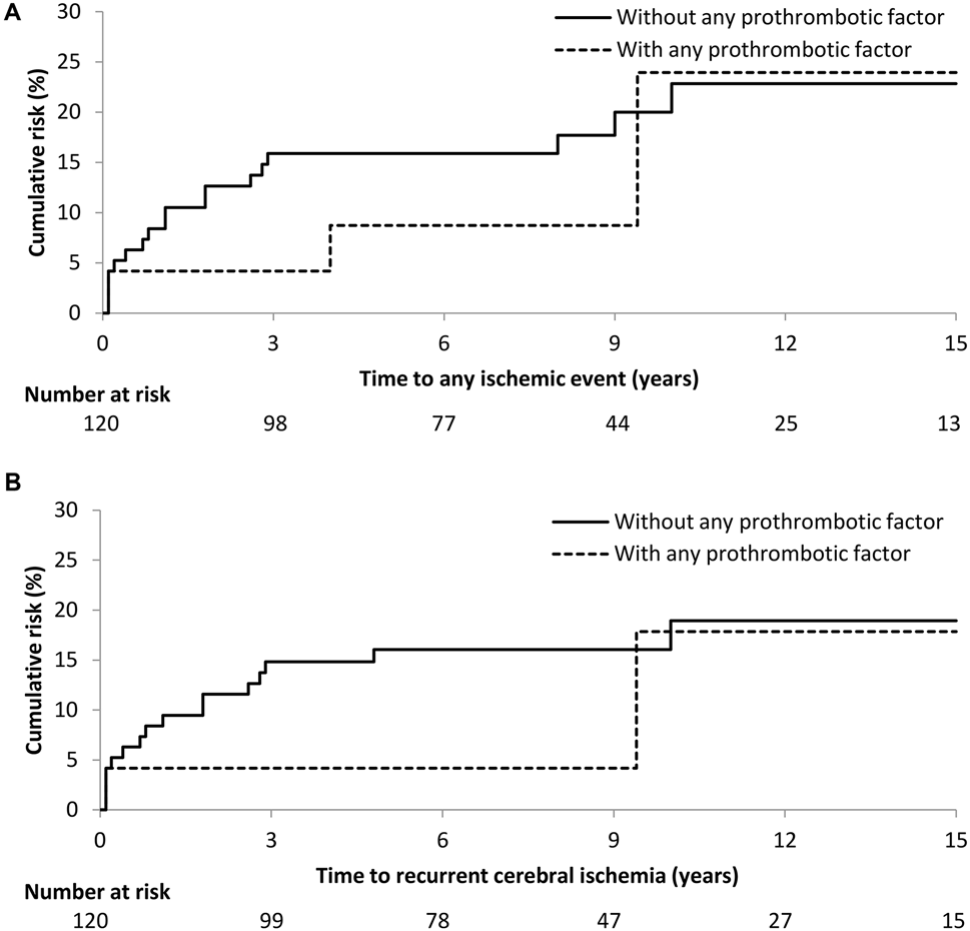
Cumulative risk of **a** any recurrent ischemic event and **b** recurrent cerebral ischemia after cryptogenic stroke in patients with and without any prothrombotic factor

There were no differences between patients with or without any prothrombotic factor with regards to their 15-years cumulative risk of any recurrent ischemic event (24 and 23% respectively; log rank p = 0.42) or cerebral ischemia (18 and 19% respectively; log rank p = 0.35) (Fig. 1), nor were there significantly differences between patients with and without any aPL with regards to their 15-years cumulative risk of any recurrent ischemic event (43 and 17% respectively; log rank p = 0.63) or cerebral ischemia (25 and 15% respectively; log rank p = 0.89).

In univariate analyses, neither the presence of an individual prothrombotic factor, a recent infection, nor any prothrombotic factor or any positive aPL was associated with any recurrent ischemic event in patients with a cryptogenic stroke (Table 3), nor was it associated with recurrent cerebral ischemia.

**Table 3:** Prothrombotic factors, recent infections and the univariate associations with any recurrent ischemic event in patients with a cryptogenic stroke

	n_recurrence_/n_positive_ (%)	n_recurrence_/n_negative_ (%)	HR (95%CI)	p-value
Prothrombotic factors				
Protein-C activity	0/3 (0)	19/108 (17.6)	–	–
Free protein-S antigen	0/3 (0)	20/107(18.7)	–	–
Antithrombin III activity	1/4 (25.0)	16/104 (15.4)	2.77 (0.36–21.21)	0.33
Factor V mutation	1/7 (14.3)	14/79 (17.7)	0.73 (0.10–5.61)	0.76
Factor II mutation	0/1 (0)	13/77 (16.9)	–	–
Factor VIII activity	0/4 (0)	6/53 (11.3)	–	–
Lupus anticoagulant	0/2 (0)	19/108 (17.6)	–	–
Anticardiolipin IgG	1/3 (33.3)	14/100 (14.0)	1.57 (0.20–12.48)	0.67
Anticardiolipin IgM	2/4 (50.0)	13/98 (13.3)	3.13 (0.70–13.94)	0.14
Any prothrombotic factor	3/24 (12.5)	19/96 (19.8)	0.61 (0.18–2.07)	0.43
Any aPL	2/7 (28.6)	18/110 (16.4)	1.44 (0.33–6.24)	0.63
Infectious pathogens				
Borrelia IgG	0/1 (0)	13/97 (13.4)	–	–
Borrelia IgM	1/2 (50.0)	12/96 (12.5)	5.89 (0.75–45.93)	0.09
Syphilis	1/1 (100)	12/99 (12.1)	24.09 (2.69–215.57)	0.004

HRs (95% CI) and p-values were obtained by univariate Cox proportional hazards analyses

*HR* hazard ratio; *CI* confidence interval; *aPL* antiphospholipid antibody

## Discussion

We showed that the prevalence of hypercoagulable states and recent preceding infections with Borrelia or Syphilis did not significantly differ between patients with a stroke at a young age with cryptogenic cause after a routine evaluation and in patients with an identifiable cause. In addition, neither the presence of a prothrombotic factor nor the presence of any of these two recent infections significantly increased the risk of any recurrent ischemic event or recurrent cerebral ischemia in patients with a cryptogenic stroke after a mean follow-up of 8.9 years. One of the strengths of our study is the large number of young stroke patients, including those with an unknown cause after routine evaluation, with data from a single centre. Thereby, it is the only study with a long-term follow-up that allows investigating the risk of recurrent ischemic events, depending on the presence of a prothrombotic factor or a recent infection. However, some limitations need to be addressed. First, statistical power was limited because of the low prevalence of a prothrombotic factor or a recent infection. As a consequence, we cannot rule out that the absence of an increased risk of recurrent ischemic events is a false negative finding due to lack of statistical power. Second, due to the changes in standard diagnostic procedures over time data on prothrombotic factors and recent infections were not complete in all patients. This may lead to an underestimation of the total number of patients with a prothrombotic factor. Nevertheless, in 77% of the included patients with a cryptogenic stroke at least 1 prothrombotic factor was determined. Third, positive results were not confirmed, so false positive results due to an acute phase response could confound the results. Fourth, non-participants might have been those with a worse outcome than participants. This could potentially lead to an attenuation of the risk of cerebral ischemia associated with prothrombotic factors and infectious pathogens in young patients. However, there were no differences in baseline characteristics.

Prior research that investigated the role of the five most inherited coagulation disorders (protein C deficiency, protein S deficiency, antithrombin III deficiency, factor V mutation and factor II mutation) as risk factors in young ischemic stroke patients did not show unanimous results. The majority of the case-control studies did not provide evidence for an association of these coagulation disorders and stroke. However, some found a positive relation between factor V or factor II mutation and ischemic stroke [4–6]. We found a non-significant higher prevalence of heterozygous factor V mutation in patients with a cryptogenic stroke (8.1% vs. 2.5%), but this was not associated with an increased recurrent stroke risk.

An increased activity of factor VIII was found in 7.0% of our patients with a cryptogenic stroke. The relationship of increased factor VIII activity with ischemic stroke has not been extensively studied and especially not in young adults. In contrast to our results, the study of Pahus et al. [28] showed that none of the young patients with ischemic stroke had an increased factor VIII activity, although factor VIII activity was operationalized as having persistent increased levels. Therefore, their proportion of patients is probably lower due to regression towards the mean and due to factor VIII activity being an acute-phase response protein.

The prevalence of positive aPLs varies tremendously across studies. In a systematic review based on data from 43 studies among young stroke patients the median frequency of any positive aPL was 17% (range 2–56) [7]. If only the 13 studies with confirmed aPL positivity, at least 6 or 12 weeks apart were included, still a median frequency of 17% (range 2–55) was found. Furthermore, the presence of any positive aPL conferred a fivefold increased risk for stroke compared to controls. The median frequency in this review is higher than the prevalence of any positive aPL we found (6%), even though we only measured all aPLs once. Moreover, the prevalence of any positive aPL in our patients is in accordance with a large recent study of Pezzini et al. [10], who found persistently positive aPLs in 110 (6.4%) of their young stroke patients.

In our study, a recent infection with Borrelia or Syphilis was rare. Neuroborreliosis and syphilis have been associated with stroke in young adults without traditional vascular risk factors [29, 30]. However, data that support an association between these infections and stroke are scarce and are mostly derived from case series [31]. We therefore recommend that testing for these infections should only be considered in patients without other obvious causes of stroke and with clinical symptoms suspicious for a recent infection [29, 31].

In our study, none of the prothrombotic factors individually nor the presence of any prothrombotic factor was associated with an increased risk of any recurrent ischemic event or recurrent cerebral ischemia in patients with a cryptogenic stroke. Compared to other studies, we used a very selected group of patients with an unknown cause of their stroke after standard evaluation with less potential sources of confounding. However, this selection reduces the statistical power. Our results are in accordance with Munts et al. [12], who did not find that the presence of any coagulation disorder was associated with an increased risk of recurrent thrombotic events in young adults after cerebral ischemia. Pezzini et al. [10] reported that the long-term risk of recurrent thrombotic events after ischemic stroke at a young age was attributable to positive aPLs (HR 2.4), but not to factor V or factor II mutations. One other study reported that young adults with cerebral ischemia and positive aPLs were more at risk to develop recurrent ischemic events,[14] although another did not demonstrate such an association [11]. Our study did not find the presence of any positive aPL to be related with recurrent ischemic events in patients with a cryptogenic stroke. However, only 7 patients with any positive aPL were identified.

A convincing recent systematic review shows some evidence that positive aPLs are associated with an increased risk of cerebral ischemia at a young age when compared to healthy controls [7]. Besides, some studies reported that the risk of recurrent ischemic events is attributable to any positive aPL. Therefore, large long-term prospective studies are essential to better assess the role of positive aPLs as risk factors for cerebral ischemia in young adults. Other prothrombotic factors probably do not seem to play a major role in the pathogenesis of cerebral ischemia in the young. As a consequence, the treating physician should only screen for antiphospholipid antibodies in patients with a cryptogenic stroke after standard evaluation and may consider adding other tests if there is a specific clinical suspicion. This will substantially reduce the laboratory testing expenses.

In conclusion, we have shown that among patients with a stroke at a young age the prevalence of a hypercoagulable state or a recent infection with Borrelia or Syphilis did not significantly differ between patients with a cryptogenic stoke and indentified cause of stroke. In patients with a cryptogenic stroke prothrombotic factors probably are not associated with an increased risk of recurrent ischemic events. Future studies should be large enough not only to study the relation between protrombotic factors and incident stroke, but also its interaction with for example a cardioembolic cause and the subsequent risk of stroke.

## Electronic supplementary material

Below is the link to the electronic supplementary material.

Supplementary material 1 (PDF 239 KB)
