# Supplementary material for: Prothrombotic factors do not increase the risk of recurrent ischemic events after cryptogenic stroke at young age: the FUTURE study
Source: J Thromb Thrombolysis. 2018 Feb 26;45(4):504–11. doi: 10.1007/s11239-018-1631-4 (PMC5889776; doi:10.1007/s11239-018-1631-4)
Supplement: Supplementary file 1 — Supplementary material 1 (PDF 239 KB) [file 11239_2018_1631_MOESM1_ESM.pdf]

## ONLINE SUPPLEMENT

# Prothrombotic factors do not increase the risk of recurrent ischemic events after cryptogenic stroke at young age
**The FUTURE Study**

M.M.I. Schellekens[*] MD; M.E. van Alebeek[*] MD; R.M. Arntz[*] MD, PhD; N.E. Synhaeve†, MD, PhD; N.A.M.M. Maaijwee‡, MD, PhD; H. Schoonderwaldt MD, PhD, M.J. van der Vlugt§ MD, PhD; E.J. van Dijk[*] MD, PhD; L.C.A. Rutten-Jacobs¶, PhD; F.H. de Leeuw[*] MD, PhD

Corresponding author: Professor Frank-Erik de Leeuw
RadboudUMC, department of Neurology, PO Box 9101, 6500 HB Nijmegen, the Netherlands
T: +3124-3616600 F: +3124-3618837
FrankErik.deLeeuw@radboudumc.nl

* Radboudumc, Donders Institute for Brain, Cognition and Behaviour, Center for Neuroscience, department of Neurology, PO Box 9101, 6500 HB Nijmegen, the Netherlands
† Department of Neurology, Elisabeth Tweesteden Hospital, PO Box 90151, 5000, LC Tilburg, the Netherlands
‡ Center for Neurology and Neurorehabilitation, Luzern State Hospital, Spitalstrasse 31, 6000, Luzern 16, Switzerland.
§ Radboudumc, department of Cardiology, PO Box 9101, 6500, HB Nijmegen, the Netherlands
¶ University of Cambridge, Department of Clinical Neurosciences, Cambridge, United Kingdom

# Supplemental Table

**Supplementary Table I** Distribution of prothrombotic factors and recent infections stratified by TOAST category.

| | Positive result/$n_{investigated}$ (%) | | | | | | | |
|---|---|---|---|---|---|---|---|---|
| | Total | Large artery disease | Likely large artery disease | Cardio-embolic stroke | Small vessel disease | Other defined | Multiple causes | Unknown |
| Prothrombotic factors | | | | | | | | |
| Protein C activity | 7/255 (2.7) | 1/10 (10.0) | 0/41 (0) | 1/25 (4.0) | 1/32 (3.1) | 1/39 (2.6) | 1/8 (12.5) | 2/100 (2.0) |
| Free protein S antigen | 6/252 (2.4) | 0/10 (0) | 1/41 (2.4) | 0/26 (0) | 0/32 (0) | 1/37 (2.7) | 1/7 (14.3) | 3/99 (3.0) |
| Antithrombin III activity | 15/262 (5.7) | 1/10 (10.0) | 3/42 (7.1) | 2/34 (5.9) | 2/32 (6.3) | 4/37 (10.8) | 0/8 (0) | 3/99 (3.0 |
| Factor V mutation | 10/207 (4.8) | 0/7 (0) | 0/30 (0) | 0/28 (0) | 2/29 (6.9) | 6/34 (17.6) | 0/3 (0) | 2/76 (2.6) |
| Factor II mutation | 3/189 (1.6) | 0/6 (0) | 0/29 (0) | 1/27 (3.7) | 1/24 (4.2) | 0/29 (0) | 0/3 (0) | 1/71 (1.4) |
| Factor VIII activity | 10/157 (6.4) | 0/4 (0) | 1/29 (3.4) | 2/23 (8.7) | 3/19 (15.8) | 1/26 (3.8) | 0/4 (0) | 3/52 (5.8) |
| Lupus anticoagulant | 7/262 (2.7) | 0/10 (0) | 1/40 (2.5) | 1/32 (3.1) | 0/32 (0) | 3/39 (7.7) | 1/9 (11.1) | 1/100 (1.0) |
| Anticardiolipin IgG | 6/249 (2.4) | 0/10 (0) | 1/36 (2.8) | 0/34 (0) | 1/30 (3.3) | 0/39 (0) | 1/6 (16.7) | 3/94 (3.2) |
| Anticardiolipin IgM | 7/248 (2.8) | 0/10 (0) | 1/36 (2.8) | 1/34 (2.9) | 0/30 (0) | 0/39 (0) | 1/6 (16.7) | 4/93 (4.3) |
| Any prothrombotic factor | 56/294 (19.0) | 1/10 (10.0) | 6/45 (13.3) | 7/40 (17.5) | 8/35 (22.9) | 14/46 (30.4) | 3/9 (33.3) | 17/109 (15.6) |
| Any aPL | 17/284 (6.0) | 0/10 (0) | 3/40 (7.0) | 2/37 (5.4) | 1/34 (2.9) | 3/44 (6.8) | 2/9 (22.2) | 6/107 (5.6) |
| Infectious pathogens | | | | | | | | |
| Borrelia IgG | 3/239 (1.3) | 0/12 (0) | 1/36 (2.8) | 0/28 (0) | 1/30 (3.3) | 0/42 (0) | 0/4 (0) | 1/87 (1.1) |
| Borrelia IgM | 5/239 (2.1) | 0/12 (0) | 1/36 (2.8) | 0/28 (0) | 1/30 (3.3) | 1/42 (2.4) | 0/4 (0) | 2/87 (2.3) |
| Syphilis | 1/249 (0.4) | 0/13 (0) | 0/38 (0) | 0/30 (0) | 0/30 (0) | 0/41 (0) | 0/6 (0) | 1/91 (1.1) |

Abbreviations: TOAST, Trial of ORG 10172 in Acute Stroke Treatment; aPL, antiphospholipid antibody.
